# The “Happy Heart” educational program for changes in health habits in children and their families: protocol for a randomized clinical trial

**DOI:** 10.1186/s12887-015-0336-5

**Published:** 2015-03-10

**Authors:** Vanessa Minossi, Lucia Campos Pellanda

**Affiliations:** Post-Graduation Program in Health Sciences: Cardiology, Instituto de Cardiologia/Fundação Universitária de Cardiologia, Lucia Campos Pellanda – Research Unit, Av. Princesa Isabel, 370, Santana, Porto Alegre, 90620-000 RS Brazil; Universidade Federal de Ciências da Saúde de Porto Alegre, Porto Alegre, RS Brazil

**Keywords:** Children, Family, Risk factors, Obesity

## Abstract

**Background:**

The prevalence of childhood obesity increased worldwide in recent decades and is associated with risk factors for the development of chronic diseases in adulthood. Strategies for health promotion directed at an early age, with recommendation for healthy habits, can achieve good results. The objective of this study is to evaluate the effectiveness of an innovative, simple and cost effective educational program to improve eating habits, physical activity and the knowledge about healthy habits in children, as well as in their families, as compared to routine outpatient care.

**Methods/Design:**

The study is designed as a randomized clinical trial. Sample size is estimated to include 37 children, aged between 7 and 11 years, and their guardians, randomized for an intervention or a control group. The intervention will consist of 11-weekly group meetings of nutritional education and distribution of explanatory material, with orientation about healthy food and family habits and physical activity. Recreational, simple and low cost resources, carefully designed for the presentation of contents to the children and parents, will be used in all meetings. The control group will receive standard outpatient care based in individual clinical practice guidelines. The primary outcomes will be changes in dietary habits, knowledge and physical activity of children and adults. The secondary outcomes will be changes of body mass index, waist circumference, systolic and diastolic blood pressure and laboratory tests, in children and adults.

**Discussion:**

The Happy Heart Study offers a playful and low-cost approach for the prevention and control of obesity and cardiovascular disease in children. Although this program is being planned for implementation in Brazil, the method can be adapted to many other countries.

**Registry of protocol:**

Protocol registered on the site ensaiosclinicos.gov.br: RBR-8ttw64.

## Background

The global incidence of childhood obesity in recent decades has been considered as a worldwide epidemic, present in all socioeconomic classes, age and ethnical groups [1]. Although this incidence has plateaued in many developed countries, it is still increasing in many parts of the world [2-4]. Children with excess weight are at a greater risk of obesity in adult life. In a cohort that included 1709 overweight 5–12 year-olds, almost half of the girls and a third of the boys became obese adults before the age of 40. Of those with more than 12 years old, 60% remained obese in adulthood [5]. A systematic review showed that overweight children had twice the risk of becoming overweight adults, compared with healthy weight children [6].

Poor lifestyle habits, such as consuming processed foods, with high sugar, fat and sodium content; reduced consumption of fruits and vegetables; sedentary activities such as long periods spent watching on TV or at the computer; all contribute to the continuing increase in the prevalence of overweight among children [7-10].

Previous studies have shown that the habit of watching more than two hours of TV a day is associated with an unfavorable body composition [10] and may affect physical and psychological health. Decreased levels of physical fitness, low self-esteem, social behavior change and lower levels of academic achievements have been related to screen time [11].

Conversely, changes in lifestyle, including decreasing screen time, reducing consumption of fat-rich foods and reducing physical inactivity may lead to decreased body mass index (BMI) [11,12].

Childhood is considered as a critical period, during which the patterns of dietary habits and lifestyle are learnt and rooted [13,14]. The fact that, in this age group, the control over eating habits is still largely dependent on the parents makes the treatment more complex, since success depends on involving the family in the whole reeducation process [11,15-17]. Results of a meta-analysis revealed that interventions that included family members produced larger effects than interventions focusing only in children [18,19].

Therapeutic groups represents another important option, since they promote and enhance the process of health education. Problems are divided among the group components and solutions are sought collectively, helping to achieve the bigger objective which is the change in behavior, both for the family and the child [17-19].

There is evidence that social support helps to improve lifestyle habits. Many patients find the motivation and energy required to maintain healthy eating plans through the support of their peers. Group support is one of the most valuable ways of support, especially regarding motivation [11].

Preventive interventions, including health education strategies to reduce obesity and other cardiovascular risk factors in children, are important tools for the reduction of the epidemics cardiovascular diseases in the future [20-22]. Educational programs focusing in food habits are being implemented in various countries [23-25], but studies on interventions to reduce cardiovascular risk factors in childhood in the routine pediatric ambulatory are still scarce [26,27].

A systematic review and meta-analysis conducted by our group showed that education and physical activity interventions in children have good results on blood pressure control and biochemical parameters [28], however, there is little effect an antropometric measures [28].

Individuals develop theories to explain experiences and discuss concepts from observations and analyzes, thus systematizing their knowledge. The information in itself is not enough to change habits and attitudes; only when the individual gives sense to the information that it becames knowledge, which can lead to an attitude change. Knowledge is processed and acquired from several ways, depending on the context, the time, the emotional status and motivation of individuals. It is noteworthy that perception is influenced by socio-cultural habits and by the personality of the perceiver, which determine how the individual sees, hears, thinks, acts and feels [21].

Scholars in the field of education explain the construction of knowledge in several ways. In accordance with the Swiss educator Jean Piaget, the development of intelligence in children is based on an approach that considers knowledge a result of subject-object interaction [29]. The subject learns by his actions, as he/she builds his knowledge from interactions with the environment. Development is an ongoing and always expanding process [29]. Russian neuropsychologist Lev Vygotsky believed that education and knowledge building take place within a cultural-historical context. Thus, knowledge building occurs through experiences and habits, attitudes and values of those who interact with the child in his family group and in their living group. Therefore, it is in the intra- and interpersonal relationships that the subject will internalize the knowledge of social roles and functions [30]. For the researchers Maturana [31,32] and Varela [33], life is a continuous process of knowledge. Educating constitutes a process during which the individuals live with one another, constantly adjusting this coexistence in a way in which makes progressively more congruent with each other in the living space. Learning happens, therefore, all the time and in a reciprocal way [31-33]. For Freire, the maturation of new possibilities and new challenges occurs through the exploitation of the environment; the subject builds new answers, and the construction of knowledge occurs through interaction with the world. The knowledge of the child in early childhood is based on the observation of the attitudes of adults. Bridging this concept to health education in childhood, playful activities are very effective in the teaching-learning process, arousing curiosity and facilitate the child’s understanding [34]. The pedagogical workshops can serve as catalysts for construction of collective/individual creative knowledge. Every workshop needs to promote search, action and reflection, combining individual and collective work and ensuring the unity of theory and practice [29,32].

A study developed in Brazil with children 5 – 10 years-old and their teachers showed that, after nutritional interventions, there was an increase in students’ knowledge from 61.1% to 73.6%; teachers also showed a significant increase in their knowledge [21]. The same was found in the 464 children, with the use of a Cognitive Theory based programme [34]. Studies based in interventions designed to increase knowledge of patients with chronic diseases showed increased knowledge and improved self-care of these individuals [35,36]. Thus, increasing knowledge may help to improve self-care related to life habits changes.

In this context, the present study aims to present a differentiated proposal that includes technological resources that are already part of the lives of young people. The target population is formed by schoolchildren, also called “digital natives”. The construction of learning in a healthy education should involve attractive tools, in accordance with the reality of the world in which they live, in order to reach a more effective practical modification of life habits [28,37,38].

This proposal aims to work with a mixture of low cost and innovative materials, associating recreational activities with everyday knowledge of the participants and scientific knowledge of professionals involved, through the formation of a group (children and parents) in which participants can share experiences [11-15]. It is a proposal involving collective construction of healthy habits, with the potential to reach a large number of people, thus reducing expenses with the consequences of future chronic diseases [22,23].

In addition, the use of a multidisciplinary educational intervention protocol for prevention of chronic diseases, guiding the child and family toward the construction of a critical, conscious and healthy thinking, can allow the individual to multiply the knowledge among family, school, work and community as a whole. After validation by the present study, this protocol may be used in different settings (school, family health strategy, community centers), allowing the prevention of chronic diseases through an educational program.

Thus, our hypothesis is that an educational program with playful activities for children and families, including text message reminders and social media composed of recreational activities is effective to increase the knowledge, physical activity and improve cardiovascular risk in children with risk factors, when compared to usual outpatient management. The objectives of the study are evaluate the effectiveness of an innovative, simple and cost-effective educational program in improving eating habits, physical activity and the knowledge of healthy habits in children, as well as their families, compared to routine ambulatory medical care.

## Methods/Design

### Study design

**Figure 1:**
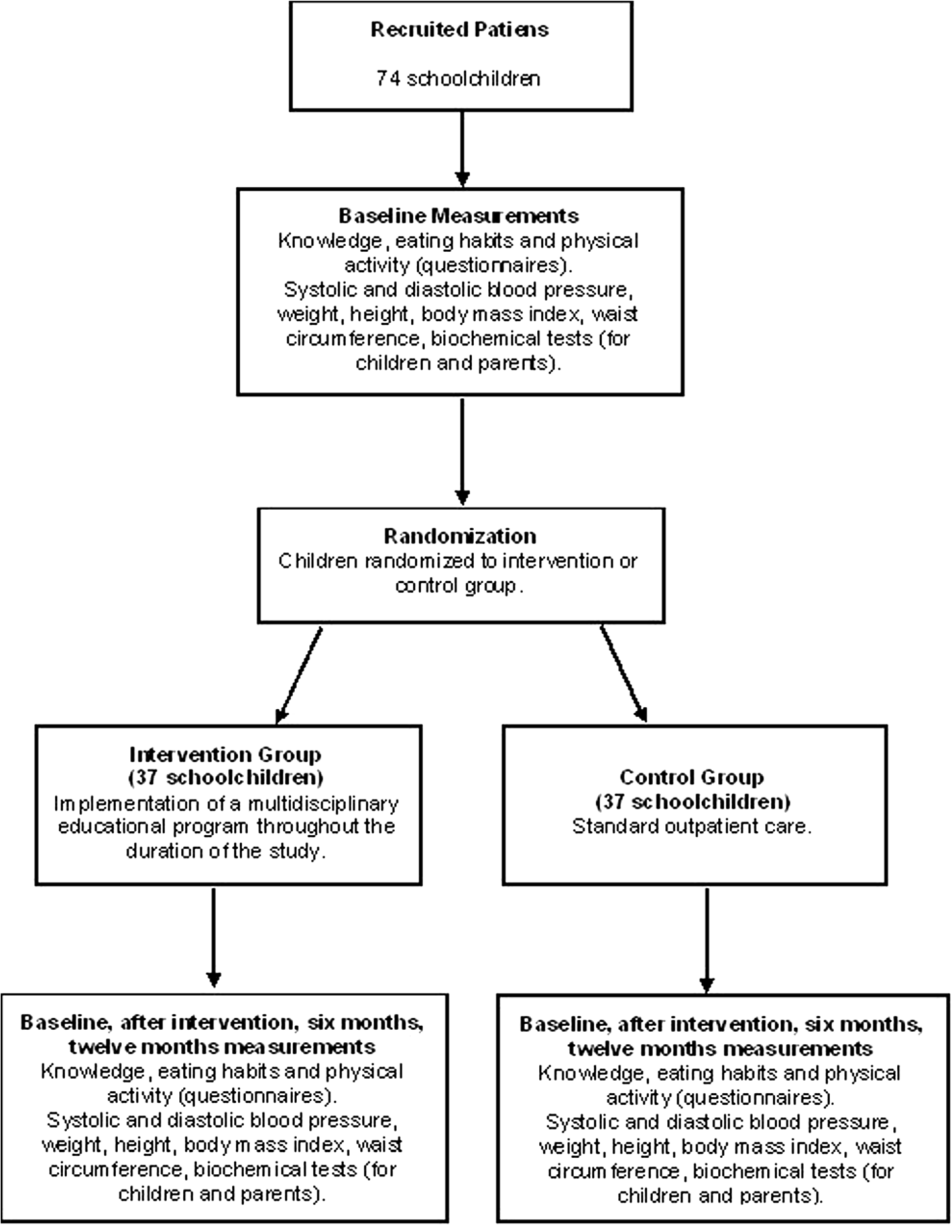
**Flow chart: study design.**

The study is planned as a randomized controlled, outcome evaluator-blinded parallel prospective clinical trial. This protocol is based in the CONSORT and SPIRIT statements for reporting of clinical trials and protocols [39,40]. A total of 74 children will be recruited. After the initial assessment, children will be randomized to either the intervention or the control groups with 1.1 allocation (Figure 1). The study setting is the “Instituto de Cardiologia do Rio Grande do Sul”, a cardiology hospital in south Brazil.

### Participants and recruitment

Patients will be recruited through public announcements in radio and newspapers of wide circulation in the State of Rio Grande do Sul, and through referrals by health professionals from the public health system. All children and their guardians will be informed about the goals and methods of the study. Parents and children who meet the inclusion criteria and agree to participate in the study will sign an informed consent.

### Inclusion/exclusion criteria

Inclusion criteria are: Children aged 7 11 years presenting at least one of the following risk factors: overweight or obesity, hypertension, dyslipidemias or diabetes.

Exclusion criteria are: contraindications for the practice of physical activity, use of drugs that would interfere with body weight and/or lipid profile and neurological or cognitive deficits that would prevent the completion of questionnaires.

### Interventions

The goal of the intervention is to improve health habits of students and their families. Children and their families will receive intervention for eleven weeks. The materials will be developed around the following key messages:

- Consequences of inadequate eating habits;
- Recommendation of adequate intake of nutrients;
- Health promotion strategies.

The materials will be designed for a fun, entertaining appearance. The meetings will be practical and playful, including culinary workshops, games and homework assignments. All materials were developed targeting this specific cultural environment, based on our 10-year work with children in the Public Health System in Brazil. We have researched materials from other countries, but we felt that developing our own materials would benefit other groups in similar situations. We also plan to involve the children in the creation of materials during the workshops. Materials are fun and playful because they are mostly based in popular games and ludical activities. Workshops will be held in 3 different days of the week, from 6 PM to 7:30 PM. The setting is a health school located in the complex of Instituto de Cardiologia, with an ample room to accommodate children and families. All the members of the family that wish to participate together with their children will be invited. Children and caretakers will attend the workshops together, except in the days when the cooking workshops will take place at the cooking lab (days 3 and 8). During the 9 weeks of workshops, text messages will be sent twice a week, on Mondays and Saturdays (total of 18 messages). Message content will be related to practical tips for a healthy lifestyle. Some examples are presented in Table 1.

**Table 1:** **Examples of text messages for families**

**Number**	**Message**
1	Remember to eat vegetables today and everyday!
2	Eat slowly and enjoy your food!
3	Avoid soft drinks; substitute for a nice up of water :-)
4	Water is important for the organism to work well; remember to take a glass now!
5	How about turning off the TV now and go for a family walk?
6	Use different vegetables. An idea: grate a red cabbage and a carrot; add an apple and season with olive oil, balsamic vinegar and black pepper.
7	The way food is introduced to kids is very important, as this may determine if he/she will accept it or not. Even when the child refuses the food, you may try another way to introduce it. Do not give up!
8	To stimulate children to practice sports is a key factor in the fight against childhood obesity and other diseases.
9	The best strategy to make a sandwich tastier, nutritive and less caloric is to increase leafs and vegetables among its ingredients. Green leafs, as lettuce, spinach and vegetables such as tomatoes, carrots, beet, green pepper and hearts of palm fill your sandwich, giving a sturdy appearance to the snack and, at the same time, promoting greater satiety.
10	Try to use less salt in your food and remove the saltshaker of the table. Avoid consuming industrialized food with lots of salt, like hamburgers, sausage, ham, pretzels, canned vegetables, and manufactured soups, sauces and seasonings.

After the end of each workshop, the participants will receive information about the importance of healthy practices for cardiovascular health and homework assignments (painting, collages, games, creating stories and drawings), in order to improve awareness and encourage the correct choice of foods to improve lifestyle. Text messages with motivational phrases will be sent to the parents during the week and, in the days prior to meetings, they will be reminded of the local and time of activities, in order to stimulate attendance. In addition, a closed group will be created on the social network Facebook, for sharing experiences, exchanging recipes, questions, progress and suggestions, thus strengthening the link between patients and professionals.

A total of 11 meetings will be held. The first and the last meetings will be used for nutritional and biochemical assessment and the nutrition education program will be developed during the remaining nine meetings with 90-minute weekly workshops, summing up a total of eleven weeks of participation. The activites will be deleveloped as part of the educational program, as shown in Table 2.

**Table 2:** **Activities to develop in the educational program**

**Meeting**	**Description of workshops**
1	Food pyramid class. The food pyramid will be explained with an acrylic pyramid and life-size food replicas. The importance of a healthy and balanced diet will also be emphasized. This concept will be illustrated with replicas of an artery for visualization of the effects of a diet rich in fats, sodium and sugar in the blood vessels. Other subjects discussed in this meeting will include the dimension of portions, plates, glasses, cups and other kitchen utensils, in order to teach about the proper amounts for the age group. Children and their guardians will have the opportunity to discuss questions and express their expectations for the educational program.
2	Discussion on the quantity of sugar, sodium and fat in foods. The workshop aims to clarify, using food kits, the quantities of these nutrients in foods most frequently consumed by the group. Children and families will also be taught how to read food labels correctly, to be able to take healthy decisions on daily basis.
	Educational materials will be used with drawings showing comparative food information, to illustrate how to make the best choice. The parents will receive two food guides, “In Search of a Happy Heart” and “Sodium”. In this same workshop, the children will build an educational whiteboard, with the objective of assisting in organizing the family routine. Parents and children will establish rules and routines. Regarding food, tidiness and order, for a healthy coexistence within the family. This whiteboard will be used at home to record their children’s daily behavior and teach them to self-assess their health habits.
	Directions for use:
	• choose the location where the whiteboard will be placed;
	• in the first column, set the activities;
	• the child will be evaluated daily on the activities performed, and earn a happy face at each accomplished task.
	• at the end of each month, the child will win or not a prize established by parents, depending on the nutritional pattterns during the month;
	• the prize will be awarded if the child wins at least 18 happy faces during this period.
	Suggestions:
	• it will be suggested to parents that the award is not focused on material objects, but in family activities such as going to parks or zoos, cycling or flying a kite, strengthening the family bond;
	• the whiteboard is intended to help the child to develop the idea that good habits result in good moments, not in gifts.
3	Healthy nutrition class in the kitchen. On this workshop, adults will work in the kitchen, along with the nutritionist, to prepare healthy snacks with vegetables and fruits, and learn to make substitutions in order to reduce the amount of salt, fat and sugar used at home.
	The children will receive a paper food pyramid to draw and paste the food in the right places. This workshop will also include a tale hour with the book *Correct Food.*, with an explanation about foods that are good or bad for the health. Finally, the workshop will be closed with the tasting of healthy snacks prepared by the parents.
4	Meeting with parents. In this meeting, the psychologist will conduct a team-building activity with discussions and analyses on what and how to do to change erroneous habits and attitudes in the family routine.
	In this meeting, the children will participate in a culinary workshop with the nutritionist and learn the proper technique of hand hygiene with the aid of a red washable ink. They will then prepare a “healthy orange soft drink”, with orange and papaya juice, as well as a whole grain cocoa cake. The children will also be taught about grilling, roasting and other food cooking techniques, aiming to help them have good eating habits. Finally, the children will receive a book of healthy recipes.
5	Physical activity. In this workshop, the physical educator will conduct games such as jump rope, elastic game, hopscotch, hula-hoop and video games (XBOX), so that the children and their parents exercise. The workshop aims at teaching easy exercises that do not demand much space and can be done at home. In this meeting, the children will receive lettuce seedlings to plant and create small gardens in their homes, as a means to stimulate the consumption vegetables.
6	Meeting with parents. The therapist will conduct a discussion about important points, such as how to establish limits and to deal with bullying and low self-esteem issues.
	The children will be distributed in teams for playing games about healthy eating, aiming to teach about food through the senses of touch, smell, taste and vision.
	**Vision:** fresh, whole or cut food will be shown;
	the children will be asked if they know each food presented and if they like it.
	**Tact:** the children will be blindfolded and the nutritionist will choose one food for each child,
	prompting her to handle and identify it.
	**Smell:** the child, still blindfolded, will be asked to smell and identify the food.
	**Palate:** the blindfolded child will receive a portion of the food and will be prompted to try and identify it.
	This meeting will also include other healthy eating games.
7	Meeting with parents and children. In this meeting, scenes of the movie *Muito Além do Peso* will be discussed.
8	Meeting with parents: Healthy nutrition in the kitchen: in this workshop, the guardians will join the nutritionist in the kitchen to prepare healthy dishes. The objective is to emphasize how to replace sodium, fats and sugars in food preparation.
	The children will play with the human board game, that encourages healthy habits and in which they themselves are the parts.
9	Meeting for questions and conversation. For parents and children who wish to participate, a video will be recorded on what they have learned during the meetings.
	In this last meeting, anthropometric and biochemical assessments, as well as blood pressure measurements, will be repeated in children and their guardians. All participant children will receive a certificate and a fridge magnet about “the ten healthy habits”.

### Control group

The participants will be seen at the Outpatient Cardiology Clinic of the Institute of Cardiology. Children in the control group will be seen every two weeks, receiving orientation about correct eating habits and the need to increase physical activity. This type of care is performed routinely, but a specific day will be reserved for these patients in the outpatient clinic.

Each child will receive a booklet with written guidelines. Targets will be set with the child, to promote a change of attitudes and motivation specifically focused on possibly inappropriate food choices detected.

At the end of the study, the intervention activities will be offered to all participants in the control group.

### Outcomes

**Figure 2:**
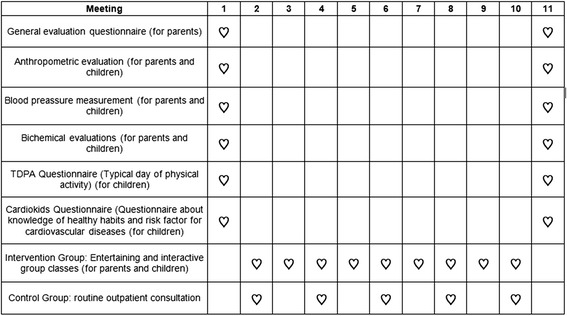
**Study timeline for intervention and assessment of outcomes.**

The primary outcomes will be the differences between the intervention and control groups with regard to changes in dietary habits, knowledge and physical activity of the participants, from the beginning to the end of follow-up for children and adults. The secondary outcomes will be the differences between the intervention and changes in body mass index, waist circumference, systolic and diastolic blood pressure, total cholesterol, LDL-cholesterol, HDL-cholesterol, triglycerides, glucose, complete blood count and C-reactive protein, both in children and their parents. Figure 2 presents a timeline for assessing the outcomes.

### Evaluation of outcomes

Time schedules for both groups is presented in Figure 2. All results will be evaluated at the beginning and at the end of follow-up, in all participants in the intervention and control groups.

A general evaluation questionnaire will be administered to the parents, to identify personal details of parents and children, breastfeeding time, age of introduction of complementary foods, early and family history. Evaluation tools used with the children will include the “TDPA” and the “Cardiokid” questionnaires.

The TDPA (Typical day of physical activity questionnaire) is an illustrated and structured questionnaire, developed in the Portuguese language by a group of researchers from Brazil, with the purpose of obtaining information about a typical day of physical activity [41]. The questionnaire was developed for children aged 7 10 years and is evaluated by a sum of scores: children reaching a total lower than 36 classified as “less active”, from 37 to 58 as “intermediate”, and from 59 to 141 classified as “most active” [41].

The Cardiokid questionnaire was developed in the portuguese language with the aim of evaluating knowledge about healthy habits and risk factors for cardiovascular disease [42]. It presents 12 illustrated and colourful questions, with three alternative answers represented by faces: “smiling” (good for the heart), “sad” (bad for the heart) and “neutral” (I don’t know). The sum of right answers determines the level of knowledge: “excellent knowledge” (11 to 12), “good knowledge” (eight to 10) and “insufficient knowledge” (below seven correct answers) [42].

The blood pressure will be measured with the appropriate size cuff. Three readings, each about two minutes apart, will be taken on the right arm, in a sitting position, with the arm supported at heart level, after a 10 minutes rest. The average of the last two measurements will be used for the analysis. For children, the systemic blood pressure will be considered abnormal if the systolic and/or diastolic measurement is greater than or equal to the 95th percentile for gender, age and height percentile [42-45]. For adults, values of systolic blood pressure ≥ 140 mmHg and/or diastolic blood pressure ≥ 90 mmHg [46] will be considered abnormal.

Body weight and height will be measured in duplicate by one of the researchers. Patients (children and parents) will be asked to remove shoes and heavy coats for this measurement. Nutritional status will be assessed by calculation of body mass index (BMI), with use of the Anthro Plus ® program, according to the guidelines of the World Health Organization [47,48]. BMI cut-off points for defining overweight and obesity in children will be percentile > 85 and percentile > 95, respectively, according to WHO-2006/2007 [49]. For adults, the BMI will be obtained through the relationship between total body weight in kilograms divided by height in meters squared, and will be classified according to the World Health Organization - eutrophy (BMI between 18.5 and 24.9 kg/m^2^), overweight (between 25 and 29.9 kg/m^2^), obesity I (between 30 and 34.9 kg/m^2^), obesity II (between 35 and 39.9 kg/m^2^) and obesity III (≥40 kg/m^2^) [50,51].

For children and adults, waist circumference will be measured with a measuring tape at the midpoint between the last palpable rib and the top of the iliac crest, where there is a greatest amount of visceral tissue. In children, waist circumference will be classified according to the National Health and Nutrition Examination Survey, 2003–2006 (NHANES 2003–2006), with cut-off points in the 90th percentile, according to gender and age. For adults, waist circumference cut-off points for increased cardiovascular risk will be considered as ≥94 cm for men and ≥80 cm for women, according to the World Health Organization [52].

Blood will be collected for analysis of total cholesterol, LDL-cholesterol, HDL-cholesterol and triglycerides, glucose, complete blood count and C-reactive protein after a 12 h fasting. The biochemist performing the assay will be blinded to the identity of the participants. Analyses will be conducted with an automatic enzyme technique, with Roche® kits (Brazil).

Lipid profile abnormalities will be defined as: total cholesterol ≥ 200 mg/dL; LDL-cholesterol ≥ 130 mg/dL; HDL-cholesterol < 40 mg/dL; triglycerides ≥ 100 mg/dL (age 0–9 years) or ≥ 130 mg/dL (age 10–19 years). Glucose levels will be considered abnormal as ≥ 100 mg/dL [43-45]. All the above cut-off points, which are very similar to the cut-off points of the Brazilian guidelines [44], were proposed in the Expert Panel on Integrated Guidelines for Cardiovascular Health and Risk Reduction in Children and Adolescents: Summary Report, of the American Academy of Pediatrics (AAP) [45].

For adults, lipid profile abnormalities will be defined as: total cholesterol ≥ 240 mg/dL; LDL-cholesterol ≥ 190 mg/dL; HDL-cholesterol < 40 mg/dL) and triglycerides ≥ 200 mg/dL). Glucose will be considered abnormal if ≥ 100 mg/dL [44,45].

### Sample size

Sample size was estimated considering a possible reduction of 20 g/dL and standard deviaton of 29,9 on total cholesterol in the intervention groups, similar to the observed in another Brazilian study [53]. The sample size was thus estimated as 37 patients in each group, considering a significance level of 0,05 and power of 0,80, with the aid of an online sample size calculator (Russ Lenth) USA [54]. Considering the outcome “BMI change”, to detect a standardized difference of 1,0 as observed by “Effects of a School-based Weight Maintenance Program for Mexican-American Children: Results at 2 years, with a bilateral alpha of 0,05 and power of 0,8, we estimated the need of a sample size of 17 participants per group [55].

### Randomization

The randomization will be performed with the aid of a sequence generated on the website www.randomization.com [56], after input of the total number of patients required, number of arms and number of blocks. The randomization in blocks was chosen because the total number of patients will probably not be available at the same date in the beginning of the study. If there is the need to apply the intervention in more than one small group in order to reach the total sample required, it is more appropriate the these patients will be evenly distributed regarding date of inclusion. The list will include ten random blocks, each with ten patients, aiming at ensuring equal distribution between the arms of the study. Allocation will be concealed by placing the randomized designations in white envelopes, which will be sealed and opened only after inclusion of the patient in the study. All steps described above will be undertaken by a third party researcher, not directly involved in the study.

### Data analysis

Data will be analyzed according to the intention-to-treat approach, with the Statistical Package for the Social Sciences (SPSS) version 20.0. Continuous variables will be expressed as means and standard deviations, and categorical variables, in absolute and relative frequencies. Groups will be compared with the Student’s *t* test for continuous variables and Chi-square test for categorical variables. Analysis of variance (ANOVA) for repeated measures will be used for intra and inter-group comparisons. Since this is a short duration trial and risks are considered minimal, we do not plan to perform interim analysis. The significance level adopted will be 5%.

### Project timelines

The recruitment of the participants began on October 1, 2013. Analyses are expected to be completed, with discussion of the first results, in March 2014.

### Ethics and divulgation

#### Ethics

In order to comply with the ethical issues in research, the project was submitted to the Research Ethics Committee of Institute of Cardiology – Fundação Universitaria de Cardiologia.

All participants will sign an informed consent form, in accordance to well-established practices. For that, they will be informed about the objectives of the study, and any questions will be answered by the researcher. All participants will be able to discontinue participation at any time, without the need for any explanation. The data will be under the guard of the researcher, with secrecy and confidentiality guaranteed.

After analysis, the data collected will be archived by the researcher for at least five years, and then will be destroyed by tearing and burning. At the end of the study, a final report will be sent to Instituto de Cardiologia, where it will be accessible to the participants of the program. A manuscript will be prepared for publication in scientific journals.

#### Divulgation

The results of this study will be widely publicized by presentations in lectures and by publications, aiming to contribute to improving child health in developing countries.

## Discussion

Although the vast majority of deaths attributed to cardiovascular diseases and obesity occur in adulthood, there is growing evidence that risks for these diseases begin in childhood and continue throughout life. In this context, early childhood education and care represents an special opportunity for prevention. Small changes in risk factors can have a considerable impact in improving the quality of life of the population.

The knowledge, attitudes, behaviors and skills developed through effective health programs may result in a better quality of life and empower children to make correct choices to promote the health of the individual, the family and the community [11,57]. To motivate participants and improve their adherence to these programs, some authors suggest the use of text messages (SMS) or phone calls prior to scheduled meetings [58,59]. Another important point to be considered is the relationship between health professionals and patients that help to ensure the adherence of patients to the proposals and guidelines [58-60]. The establishment of a relationship between patient and the professional team is important in helping the process of change, the awareness and capacity of resilience, even in the adoption of a new lifestyle [59,60].

Various methods are used to improve habits of children; however, studies show that educational alternatives, with recreational intervention strategies, are more efficient [44,61,62]. Recreational resources should be encouraged as supporting actors in the process, since during play the child acquires experience and knowledge, concepts and values. Playful activities, including games and toys, are effective instruments that facilitate the learning process, improving the child’s performance [63]. An innovative, low-cost protocol that can be easily applied in educational interventions for the treatment and prevention of cardiovascular diseases, using resources appropriate for the age group, can be used in multiple contexts [61,62].

The Happy Heart Study offers a playful and differentiated approach for the prevention and control of obesity and cardiovascular disease in children. Although this program is being planned for implementation in Brazil, the method can be adapted to many other countries. In developing countries, it is even more important to learn to prevent instead of dealing with the consequences of an established epidemics, and prevention depends on the implementation of health educational programs and healthy living habits.

Children are capable of quickly internalize what they learn, establishing a healthy lifestyle. They are also very able to spread this practice to the familiar context and to the society, as a whole increasing both short and long term results of preventive public health programs. The inclusion of the family in the intervention procedures was due to evidence that children’s treatment programs in which several members of the family participate are more successful than programs consisting only of food restriction. In addition, educational materials in the form of manuals will be provided for parents in some of the meetings.

The present protocol for prevention of chronic diseases is a simple and low cost tool, and can be incorporated into the school curriculum, in the community and in different contexts. It has the potential to produce beneficial health effects and thus considerably reduce public spending with health problems triggered in adulthood by overweight and physical inactivity in childhood, representing an important prevention strategy.
